# 

**Published:** 1921-06-18

**Authors:** 


### Waziristan Campaign Honours.

H.M. The King has given orders for the following appointments, on the recommendation of the Government of India, for valuable services in Waziristan: *Companion of the Order of the Star of India*: Col. C. W. Profeit, C.M.G., D.S.O., A.M.S. *Companion of the Order of the Indian Empire*: Col. T. Stodart, I.M.S.; Lieut.-Col. (Acting Colonel) C. Hudson, D.S.O., I.M.S. The King has also approved of the following reward: *To be Brevet Lieutenant-Colonel*: Major T. S. Dudding, O.B.E., R.A.M.C. *Bar to Military Cross*: Capt. W. J. S. Ingram, M.C., I.M.S. *Military Cross*: Temp. Capt. B. L. Gupta, I.M.S.; Temp. Lieut. M. G. Kini, I.M.S.; Temp. Capt. K. G. Mohile, I.M.S.; Capt. (Acting Major) D. Reynolds, R.A.M.C.

### Nursing Honours.

The King has awarded the Royal Red Cross to the following (all of the Q.A.M.N.S.I. or attached), in recognition of their valuable nursing services in connection with the Waziristan operations: *1st Class*.—Matron E. O'Sullivan, A.R.R.C.; Acting Senior Sister (Matron) M. E. Tippetts, A.R.R.C. *2nd Class*.—Sister E. Bateman; Temp. Nurses N. Hickie, E. C. Wilkinson, E. E. Ennis, F. Houston, C. S. James.

### Economising Fuel Costs.

Devonshire Hospital, Buxton, has recently fitted a Meldrum forced-draught furnace to a Cornish boiler. By this means low-grade fuel can be utilised for steam-raising.

### Progress of Appeals.

WITH reference to the appeal for £50,000 to defray the cost of the long-projected building extension at Queen's Hospital for Children, Hackney Road, Mr. Glenton-Kerr, the Secretary, informs us that £50,000 was a pre-war estimate and that it is now considered £100,000 would be required. Having regard to the fact that the hospital endowments only bring in £1,200 per annum and that the expenditure is £32,000 per annum, every effort has to be concentrated on obtaining money for maintenance, and the building project must of necessity be left in abeyance. No less than £15,000 must be obtained in donations during the current year. An open-air carnival in aid of the funds of the hospital will be held on July 16.

AT the Metropolitan Hospital energetic steps are to be taken to reduce the outstanding debt, which at present amounts to about £6,000. Mr. H. F. Rutherford informs us that the fifteenth annual matinée at the Hackney Empire on June 2 in aid of the funds of the institution was more successful than that of any preceding year, having benefited the hospital to the extent of £500. The football cup presented by the Chairman, Lord Howard de Walden, was instrumental in raising £250. The hospital, which always has made a point of supplying special accommodation for Jews, is prepared to devote an additional fourteen beds to their use, provided £1,000 is guaranteed annually to help defray the expense.

### Passing Events and Topics.

### The British Home and Hospital for Incurables.

Queen Alexandra telegraphed to the Duke of Portland on the occasion of the Garden-Party for the British Home for Incurables at Streatham, held on June 9, as follows: "I deplore the fact that the Home in which I take so deep an interest is in such serious financial difficulties. My earnest prayer is that the anxious strain which is being felt by all hospitals and charitable institutions under the voluntary system may soon be relieved. I earnestly trust that the appeal which is to be made this afternoon for funds to enable the Home to carry on unchecked its beneficent work will meet with a generous response."

### Post-Graduate Course on Infant and Child Welfare.

A Course of twelve practical demonstrations on the Management and Feeding of Infants and Young Children will be given by Dr. Eric Pritchard (Physician-in-Charge of the Infant Welfare Department) to qualified practitioners, commencing Tuesday, June 14, 1921. The demonstrations will be given on Tuesdays at 10.30 A.M. and Thursdays at 3 P.M. at the St. Marylebone General Dispensary. Opportunities will be afforded to students attending this course of visiting the Nursery Training School, 1 Wellgarth Road, Golders Green (five minutes from the Tube station), and seeing the methods there employed in dealing with infants. Tickets for the course are two guineas, and they may be obtained from the Secretary at 77 Welbeck Street, Cavendish Square, W. 1.

### Nurses' Home Badly Needed.

At the annual meeting of Governors of the Chelsea Hospital for Women, the Marquis of Londonderry deplored the fact that although, even a year ago, the building of a Home for the nurses and servants attached to the hospital was described as an urgent necessity, the home is still unbuilt, and, owing to lack of accommodation, nurses and servants have now to be housed in the hospital wards, with the result that patients are reduced by about half. The hospital has the land available as a site for the proposed Home, and £15,000 in hand towards the cost of the new building, but the original estimate of £10,000 is now £30,000. Owing to the coal strike, the festival dinner which was to have been held in May was postponed.

### Partial Medical Training in Mission Work.

On Commemoration Day at Livingstone College Sir George Makins emphasised the great value of partial medical training in everyday life, and instanced the help it had been in the work of orderlies and V.A.D.s during the recent war. If the benefit of partial medical training is so felt in a civilised country, how much more necessary must it be in the Mission-field, where a man is far from qualified medical aid? Such knowledge as can be obtained at Livingstone College, he felt, was most helpful to all missionaries. He was supported in this view by Dr. G. E. King, of the China Inland Mission, who advocated a year at Livingstone College for every missionary. With this they should be able to steer a clear course between the mistake of over-zeal in thinking they could do anything and the error of thinking they could do nothing.

### COLLEGE OF NURSING

(Limited by Guarantee.)

### LONDON CENTRE.

(Secretary, Miss BOMPAS, 7 Henrietta Street, W. 1.)

MEMBERS are reminded that the subscriptions for 1921-22 were due on APRIL 1 of this year, and their non-payment hampers the carrying on of the Centre.

### BIRMINGHAM THREE COUNTIES CENTRE.

(Hon. Sec., Mrs. GLEGG, 84 Hagley Road.)

ON TUESDAY, JUNE 14 (by kind permission of the Governors), under the presidency of Miss Musson, a meeting of College members was held in the Lecture Theatre of the General Hospital, Birmingham. On the question of the nomination of a Finance Committee to deal with the proceeds of the Scenic Fair, it was unanimously decided to invite six ladies and gentlemen prominent in the public life of the city to act on the committee in conjunction with eight members of the College. The College members selected were: Miss Musson, R.R.C. (General Hospital, Birmingham, local representative), Mrs. Boeddiker (Hon. Treasurer), Mrs. Glegg (Hon. Secretary), Miss Holloway (Walsall Hospital, Staffs), Miss Hutchinson (Coventry Hospital, Warwickshire), Miss Bodley (Selly Oak Hospital, Worcestershire), Miss Carless (Private Nursing Home, Birmingham), and Miss Jones (Nerve Hospital, Birmingham). The constitution of the proposed club was then discussed, and the Executive Committee of the Centre was instructed to draw up a draft of rules and regulations for future discussion.

### "The Cryes of London."

THE Cryes of London Fête, held at Prince's last week in the interests of women's training and employment as carried on under the auspices of the London Society for Women's Service, 58 Victoria Street, S.W., was a very cheery affair, and the daintiness of the costumes and the good looks of the indefatigable sellers and those who aided in the sideshows were much admired. The various professions of women were represented, that of nursing being in charge of Miss Lord on behalf of the National Union of Trained Nurses and of the College of Nursing, who jointly ran the Vegetable Stall. Thanks are due to the many kind friends who supplied the stock for the stall and to those who assisted in running it, and these Miss Lord and the London Centre Committee desire to make in full measure.

### Hospital Sunday and Advertising.

OUR suggestion that large firms should help in their advertisements to give publicity to the aims of Hospital Sunday has met with immediate response. Messrs. Marshall Roberts, Ltd., H. Holdron, Ltd., Oetzmann & Co., Ltd., and others have promised to help in the way suggested, and it is hoped that other firms will also lend their aid.

### Approved Sanatoria.

THE Ministry of Health has issued a list of Sanatoria and other residential institutions approved by the Minister for the treatment of persons suffering from tuberculosis and resident in England and Wales. The list, which supersedes that dated November 19, 1919, is arranged under the name of the administrative counties and county boroughs in which the institutions are situated. It gives the date on which the approval expires in each case, and the institutions where a course of training may be included are clearly indicated. It can be obtained from H.M. Stationery Office, Imperial House, Kingsway, price 4d. post free, or from any bookseller, price 3d. Its official description is Sanatoria List 10, May 9, 1921.

### Matrons' and Sisters' Appointments.

MANCHESTER ROYAL EYE HOSPITAL.—Miss Olive Blackburn has been appointed out-patient sister. She was trained at the Royal Infirmary, Sheffield. Miss M. Firth has been appointed ward sister. She was trained at St. Bartholomew's Hospital.

GALASHIELS COTTAGE HOSPITAL.—Miss May Barrie has been appointed matron. She was trained at Stobhill Hospital, Glasgow, and at Plaistow Fever Hospital. She has been matron of the Edinburgh Hospital for Women and Children.

BRITISH HOSPITAL, NAZARETH.—Miss Mary Parkinson has been appointed matron. She was trained at Sheffield Royal Hospital and at Jessop Hospital, Sheffield. She has been sister at the Manchester Victoria Memorial Jewish Hospital, school nurse under the Manchester Education Committee and the Lancashire Education Committee, matron of the Church of Scotland Jewish Mission Hospital, Smyrna, inspector of nuisances at Sheffield, and assistant superintendent of the Child Welfare Centre, Manchester. She holds the certificate of the Central Midwives Board and is a member of the College of Nursing.

SUNDERLAND MATERNITY HOME.—Miss Clara Taplin has been appointed sister. She was trained at the Spittals Hospital, Stoke-on-Trent, and at St. Mary's Hospital, Manchester, where she has been staff nurse, acting sister, and acting district sister. She has also been night sister at the Municipal Maternity Home, Middlesbrough.

GLOUCESTER ROYAL INFIRMARY.—Miss A. J. Durward has been appointed assistant matron. She was trained at the Royal Infirmary, Sheffield, has been staff nurse at the Fever Hospital, Sheffield, sister Q.A.I.M.N.S.R. at Queen Mary's Military Hospital, Whalley, Lancs, and night sister at the Royal Infirmary, Sheffield. She is a member of the College of Nursing.

### Forthcoming Events.

CROYDON GENERAL HOSPITAL.—"At Home" in the hospital grounds (if wet, in the hospital), SATURDAY, JUNE 25, from 4 to 6.

THE LONDON HOSPITAL NURSING STAFF.—Bazaar and Fête in the grounds of the hospital, WEDNESDAY, JULY 6, 1921, 11 A.M. to 9 P.M.

### Presentation.

ON the last day of the Scenic Fair at Birmingham, Miss Musson, R.R.C., matron of the General Hospital and local representative of the College of Nursing, was presented by the trained nurses of the Three Counties with flowers and a purse-bag in recognition of all she has done for them during many years' residence in Birmingham.

### Sale of Hospital Stores.

THE great sale of surplus Navy and Army medical stores was officially opened by the Hon. Sir Arthur Stanley, G.B.E., Chairman of the Joint Council of the Order of St. John and the British Red Cross Society, who mentioned that in the report of Lord Cave's Committee the advisability of co-operative buying in order to reduce expenditure is advocated. The sale is a very good example of what can be done in this direction, including a very large stock of medical stores, bought by the military authorities on a large scale and, therefore, on favourable terms. The result is that the joint societies, acting as the agents of the Disposal Board, are enabled to offer these surplus medical stores to the hospitals on even more favourable terms. Representatives of all the hospitals of the country have been invited to view the stores during the next few days, and it is believed that they will make good use of this unique opportunity.

June 18 1921] [Price Twopence

### The Hospital

**The College of Nursing (Limited by Guarantee)** 206

**RATES OF SUBSCRIPTION** (post free, payable in advance).

Three months, 3/3; six months, 6/6; twelve months, 13/-. (Should the cost of printing and paper as well as increased postage, necessitate alteration of these rates, the period paid for will be reduced proportionately on unexpired subscriptions.) **THE HOSPITAL "Index" to Volume LXIX., October 1920 to March 1921, is now ready, price 1/1 per copy, post free.** Address The Manager, THE HOSPITAL, "The Hospital" Building, 28/29 Southampton Street, Strand, London, W.C. 2.

